# Retroperitoneal access for robotic renal surgery

**DOI:** 10.1590/S1677-5538.IBJU.2016.0633

**Published:** 2018

**Authors:** Barrett G. Anderson, Alec J. Wright, Aaron M. Potretzke, R. Sherburne Figenshau

**Affiliations:** 1 Washington University School of Medicine, Division of Urology, Saint Louis, Missouri, USA

## Abstract

**Introduction and Objective:**

Retroperitoneal access for robotic renal surgery is an effective alternative to the commonly used transperitoneal approach. We describe our contemporary experience and technique for attaining retroperitoneal access.

**Materials and Methods:**

We outline our institutional approach to retroperitoneal access for the instruction of urologists at the beginning of the learning curve. The patient is placed in the lateral decubitus position. The first incision is made just inferior to the tip of the twelfth rib as described by Hsu, et al. After the lumbodorsal fascia is traversed, the retroperitoneal space is dilated with a round 10 millimeter AutoSutureTM (Covidien, Mansfield, MA) balloon access device. The following trocars are used: A 130 millimeter KiiR balloon trocar (Applied Medical, Rancho Santa Margarita, CA), three robotic, and one assistant. Key landmarks for the access and dissection are detailed.

**Results:**

177 patients underwent a retroperitoneal robotic procedure from 2007 to 2015. Procedures performed include 158 partial nephrectomies, 16 pyeloplasties, and three radical nephrectomies. The robotic fourth arm was utilized in all cases. When compared with the transperitoneal approach, the retroperitoneal approach was associated with shorter operative times and decreased length of stay (1). Selection bias and surgeon preference accounted for the higher proportion of patients who underwent partial nephrectomy off-camp via the retroperitoneal approach.

**Conclusions:**

Retroperitoneal robotic surgery may confer several advantages. In patients with previous abdominal surgery or intra-abdominal conditions, the retroperitoneum can be safely accessed while avoiding intraperitoneal injuries. The retroperitoneum also provides a confined space that may minimize the sequelae of potential complications including urine leak. Moreover, at our institution, retroperitoneal robotic surgery is associated with shorter operative times and a decreased length of stay when compared with the transperitoneal approach (2). In selected patients, the retroperitoneal approach is a viable alternative to the transperitoneal approach for a variety of renal procedures.

## ARTICLE INFO

**Available at: http://www.intbrazjurol.com.br/video-section/20160633_anderson_et_al/**

**Int Braz J Urol. 2018; 44 (Video #2): 200-1**
